# Soil fungal community structure and seasonal diversity following application of organic amendments of different quality under maize cropping in Zimbabwe

**DOI:** 10.1371/journal.pone.0258227

**Published:** 2021-10-14

**Authors:** Tonny P. Tauro, Florence Mtambanengwe, Shensi Mpepereki, Paul Mapfumo

**Affiliations:** 1 Department of Soil Science & Environment, University of Zimbabwe, Mount Pleasant, Harare, Zimbabwe; 2 Department of Natural Resources Management, Marondera University of Agricultural Sciences & Technology, Marondera, Zimbabwe; Feroze Gandhi Degree College, INDIA

## Abstract

Recent advocacy for Integrated Soil Fertility Management (ISFM) in smallholder farming systems in east and southern Africa show substantial evidence of increased and sustained crop yields associated with enhanced soil productivity. However, the impact ISFM on soil fungi has received limited attention, yet fungi play key roles in crop growth. Following total soil DNA extraction with ZR soil microbe miniprep kit, illumina sequencing was used to, examine the fungal communities (ITS1F) under a maize crop following co-application of organic nutrient resources including *Crotalaria juncea*, cattle manure and maize stover with inorganic fertilizers at three-time periods (T1-December, T2-January, and T3-February) in Zimbabwe. Ninety-five fungal species were identified that were assigned to *Ascomycota* (>90%), *Basidiomycota* (7%) and *Zygomycota* (1%). At T1, *Ascomycota* and *Basidiomycota* were identified across treatments, with *Ascomycota* attaining > 93% frequency. Fungal succession was noted and involved reduction of *Ascomycota* coupled by increase in *Basidiomycota* under the different treatments. For example at T3, *Basidiomycota* increased to 34% while *Ascomycota* declined to 66% under manure but remained unchanged in other two organics. Pre-season mineral nitrogen (N) associated with the ‘Birch effect’ apparently influenced the fungal community structure at T1 while readily available fertilizer N was critical at T2 and T3. The low-quality maize stover promoted the presence of *Exophiala sp SST 2011* and this was linked to N immobilization. The impact of N addition was more pronounced under medium (manure) to low-quality (maize stover) resources. Fungi required phosphorus (P) and N for survival while their proliferation was dependent on substrate availability linked to resource quality. Interactive-forward test indicated that soil available P and N were most influential (P < 0.05) factors shaping fungal communities. Co-application of medium to high quality organic and inorganic resources show promise as a sustainable entry point towards enhancing belowground fungal diversity critical in driving nutrient supply.

## 1 Introduction

Global demand for food has seen massive land-use change which, coupled with intensive utilization of inorganic fertilizers, has caused habitat destruction and management -induced soil degradation [1–3]. Degradation is generally quantified based on nutrient mining, soil erosion, nutrient leaching, soil acidification, and/or reduction in soil organic matter [3–5] with little attention paid to microbial diversity. Natural undisturbed environment offers the best habitat for microbial diversity from the substrate quality and availability, cover, space, aeration and moisture provision. Under both natural and disturbed ecosystems, soil microbes are critical in facilitating soil nutrient availability through decomposition, biological nitrogen fixation (BNF), nutrients solubilization, and stimulant production [6]. Inclusion of organic resources in integrated soil fertility management (ISFM) has shown potential in reducing soil degradation while increasing maize yields [7, 8]. However, most ISFM research tends to focus on physical and chemical benefits of organic nutrient resources and consequent impact on yield improvement with little focus on soil microbial dynamics. Furthermore, both bacteria and fungi constitute a vital component of the soil microbial community, yet more research has been directed toward bacteria than fungi [9, 10].

Fungi are a diverse group of microbes which play key roles in soil during decomposition of organic matter and nutrient cycling and recycling processes [11]. The high cost of mineral fertilizers has increased the reliance on organic nutrient resources as a fall-back mechanism by both urban and communal smallholder farmers in Zimbabwe which, when available, are combined with small amounts of mineral fertilizers to ensure food security [12, 13]. However, there is limited information on soil fungal dynamics following co-application of organic and inorganic nutrient resources in Southern Africa. Simultaneously, fungal community composition may be a soil quality indicator [14], yet there is scarcity of such information in the region. Such knowledge on fungal dynamics will promote better management of the available nutrient resources in ways that increase crop productivity while maintaining soil fungal diversity. Soil fungal diversity has been estimated to ~ 80 500 operational units or ~ 1.5 million fungal species under natural systems in the world [15]. However, only 5–13% of the global fungal species have been fully characterised [16] due to difficulties in identification, isolation, and culturing processes. The impact of different quality organic nutrient resources or applied mineral fertilizers on fungi under integrated soil fertility management (ISFM) remain an unknown in most parts of sub-Saharan Africa (SSA). Furthermore, while crop productivity in the region is affected by acute soil acidity and poor P availability, [17–19] there is paucity of work primarily focusing on factors controlling fungal diversity.

Early work on fungi used a combination of serial dilution with Rose Bengal Agar medium plus streptomycin for population analysis, isolation, and general characterization [20]. However, not all fungi could be isolated and or cultured leading to underestimation of community diversity. Use of phospholipid fatty acid (PLFA) and fatty acid methyl esterase (FAME) analytical approaches in fungal quantification was an improvement but they only identified broad groups than to species levels which was a major drawback. Further improvement came with the use of sequencing of ribosomal genes that have a fungal taxonomical internal transcribed spacer and large ribosomal subunits. Widespread use of these improved methods was apparently limited by the size of the library of clones. To reduce the cost, time and improve on the quality of the results illumina sequencing approach is utilized, which is based on sequencing-by-synthesis and reversible dye-terminator capable of selecting single bases as they are introduced on DNA strands [21, 22]. The whole process in this next-generation sequencing is split into library preparation, cluster generation and high throughput sequencing stages [23]. Here, we used the next-generation sequencing approach which offers more detail as multiple strands are sequenced with a single run [22] to examine fungal communities under ISFM. This study aimed to: (1) determine the influence of co-application of different quality organic and inorganic nutrient resources on fungi diversity within a season; (2) evaluate the differential response of fungal species and diversity to P and N application under the systems; and (3) identify soil factors underpinning changes in fungal community structure under ISFM innovations.

## 2 Material and methods

### 2.1 Study site, biomass generation and experimental design

The study was based on a long-term experiment established in 2002/03 season under the project ’*Managing soil organic matter for improved nutrient use efficiency on smallholder farms in Zimbabwe—NUESOM*’ [5, 24, 25] at Domboshawa Training Centre Domboshawa is located approximately 30 km northeast of Harare, Zimbabwe’s capital city (17°36ʹ S; 31°08ʹ E; 1542 m a.s.l.), and is in agroecological region (NR) II receiving >800 mm of rainfall annually between November and April [26]. The soils are sandy clay loams broadly classified as Lixisol [27] derived from granite, and are generally acidic, with low carbon, N, and P contents.

Three organic nutrient resources namely *Crotalaria juncea* (hereafter *Crotalaria*), cattle manure (hereafter manure), and *Zea mays* stover (hereafter maize stover) representing, high, medium and low-quality resources, respectively were selected for the study. *Crotalaria* biomass was generated by chopping shade-dried crop to 10-15cm pieces, from a crop harvested at 50% flowering. Similarly, maize stover collected from nearby fields received similar treatment prior to application. On the other hand, the manure was collected from pens without any bedding material at the Centre’s Livestock Unit. The quality of the organic resources remained unchanged across thirteen seasons (Table 1).

**Table 1:** Chemical quality attributes of different organic resources used in experiment.

Attribute	*Crotalaria juncea*	Cattle manure	Maize stover
Nitrogen (g kg^-1^)	44	9	6
Lignin (g kg^-1^)	32	83	11
Polyphenols (g kg^-1^)	30	2	295
C/N ratio	10	31	69

Adopted from [5]

The main experiment is underpinned on repeated co-application of different quality organic resources and inorganic fertilizers with the view of building soil organic matter [28], increasing crop N availability and building soil fertility of degraded soils in the short and long term [12, 29]. All three organic resources generated were repeatedly applied into soil at 4.0 t C ha^-1^ in plots measuring 12 m × 6 m which served as main plots since 2002/3 cropping season to 2015/16. Incorporation was done to a depth of 0.15–0.20 m using a hoe between November to early December before the onset of rains [30]. An additional main plot without any organic resources applied was created (hereafter referred to as control). Using basal compound fertilizer with 32% P_2_O_5_:16% K_2_O: 5% S, phosphorus, potassium, and sulphur were applied at 16.0, 14.7 and 4.6 kg ha^-1^, respectively to all the main plots [29] to maize (*Zea mays* L.) the test crop. Ammonium nitrate (NH_4_^+^NO_3_^-^) (34% N) was three splits applied to attain 120 kg N ha^-1^ to sub-plots receiving mineral N.

### 2.2 Assessing soil microbial diversity

**Fig 1:**
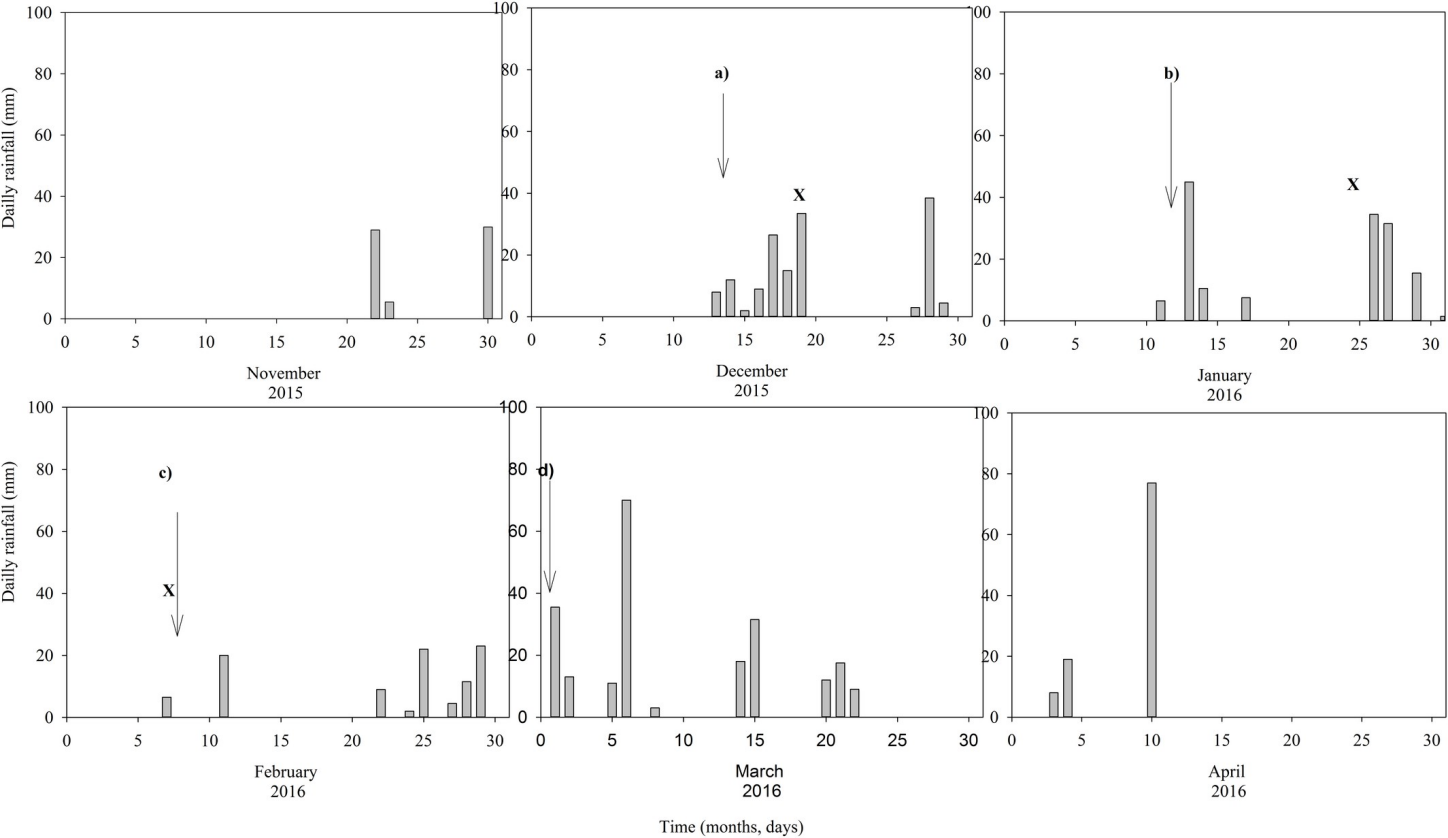
Rainfall distribution during the 2015/16 season at Domboshawa (total = 807 mm). (Major events are indicated by arrows (a: co-application of organic and inorganic nutrient resources; b: 1^st^ mineral N split; c: 2^nd^ mineral N split; d: 3^rd^ mineral N split) while bacterial sampling event by X which corresponded to 10, 36 and 49 days after incorporation of organic resources and basal fertilizers).

Composite soil samples (0–30 cm) under *Crotalaria*, manure, maize stover, and control treatments with or without N were collected in December 2015 (T1), January 2016 (T2), and February 2016 (T3) (Fig 1). Samples from the three blocks were used for pH and available P analysis but for microbial assessment they were combined and homogenized to make a composite sample at each time point [31]. Microbial samples were stored in the freezer below -20°C before being analyzed.

### 2.3. Chemical analysis of the soil samples

Soil samples were air dried and sieved to pass through a 2 mm sieve before being analyzed for available soil P using the Olsen method [32], and pH using the CaCl_2_ method [33]. Standards were freshly prepared and samples were replicated during the analysis as specified in the methods.

### 2.4 DNA extraction

Total soil DNA was extracted from 0.25 g of soil using a ZR Soil Microbe DNA MiniPrep kit (Zymo Research, Irvine, CA, USA), according to the manufacturer’s instructions. Soil samples were added to a ZR Bashing Bead Lysis Tube where microbes are rapidly and efficiently lysed by bead beating in a uniquely designed lysis buffer. The Fast-Spin column technology was then used to isolate the DNA which is subsequently filtered to remove humic acids/polyphenols that inhibit PCR. Before subjecting the DNA to sequencing, preliminary assay test we conducted to check for the presence of fungi (ITS-status).

### 2.5 18S rRNA gene sequencing

Samples were sequenced on the MiSeq system by illumina (www.illumina.com) at Inqaba biotec in South Africa. This method can sequence multiple strands and produce results faster due to automation while cost of enzymes required by pyrosequencing is eliminated [21]. Readings were processed through usearch (https://drive5.com/usearch) and taxonomic information was determined based on ITS1F, the RDP ITS V2 database. Operational Taxonomic Units (OTUs) contributing less than 1% of the total data was excluded. The frequency was calculated using read count and the total read count to provide a percentage of the OTUs at different classification levels.

### 2.6 Statistical analysis

Shannon-Wiener (Hʹ) alpha diversity index [34] was calculated in Paleontological Statistics (PAST) package version 4.02 [35]. The general linear model (GLM) with repeated measures procedure was used to separate treatments, N application and seasonal time effects on species population, taxa and diversity indices. To examine the differences in species complexity between samples, Unweighted Pair Group Method with Arithmetic Mean (UPGMA) together with the Euclidean similarity index was used for the paired group clustering analysis. All mean comparisons were considered at P <0.05 significance. Multivariate analysis (MVA) techniques were used to establish relationships among fungal species, environmental factors, and management inputs using CANOCO 4.5. Data was subjected to gradient analysis and the gradient was 4.4 which then identified unimodal pathway and detrended correspondence analysis DCA (CA) as an appropriate technique to use [36]. Management variables and quality parameters that aligned with axes were considered to have strong effects on species composition. Furthermore, an interactive-forward test was done to identify the most significant environmental factors explaining the results from DCA analysis.

## 3 Results

### 3.1 Changes in available P and pH over time

**Fig 2:**
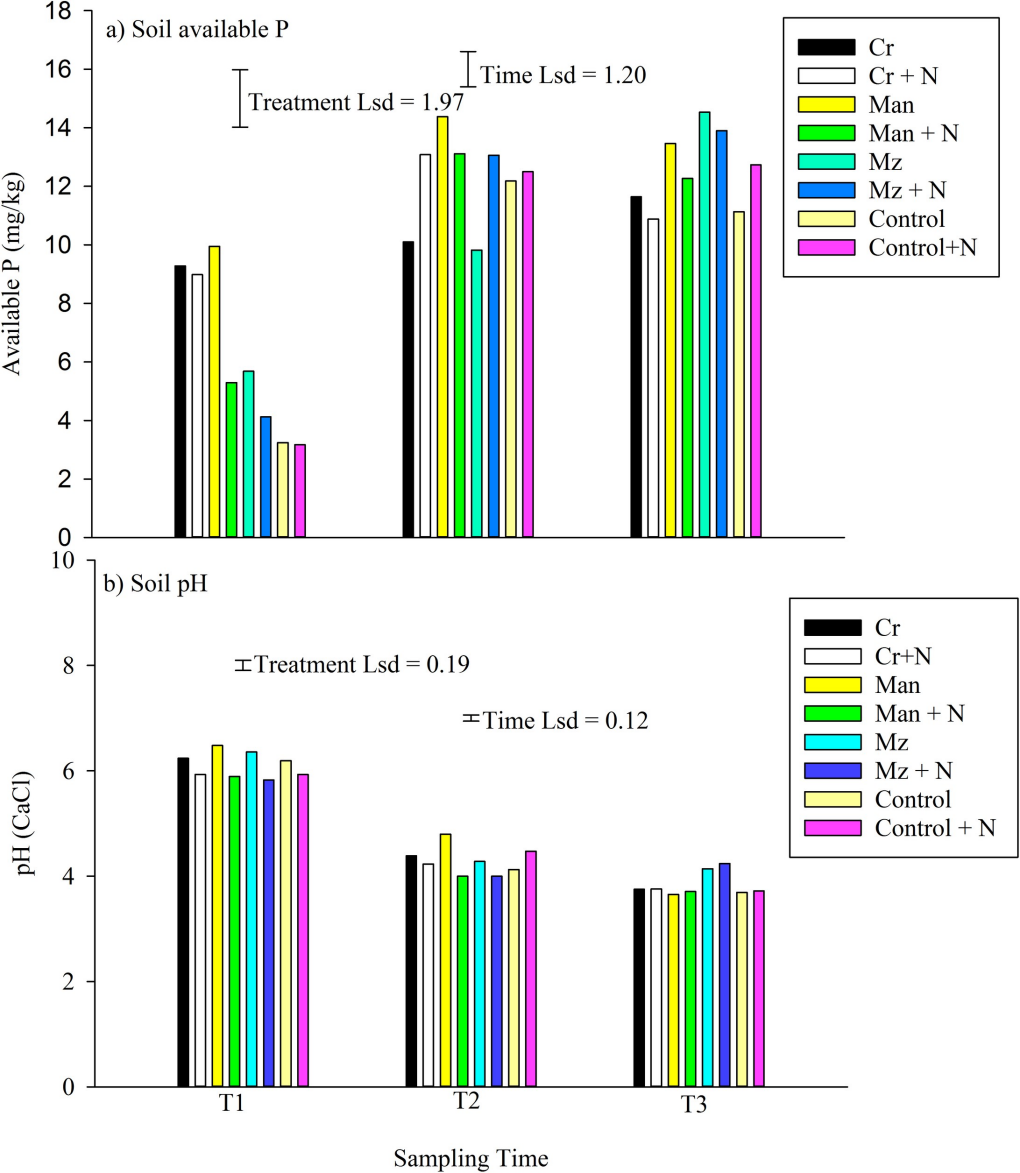
Seasonal changes in soil available P and pH at Domboshawa in 2015/16 season.

A general rise in available P was noted across treatments. At T1, the control had the lowest soil available P averaging 3.23 mg/kg while the highest (9.95 mg/kg) was under manure. Soil available P increased from T1 to T2 across treatments. At T3, maize stover with and without mineral N had the highest soil available P of > 13.9 mg/kg while the lowest soil available P of ~10.88 mg/kg was noted under *Crotalaria* + N. There was a general decrease in soil pH from T1 to T3 for all treatments with or without mineral N. At T1, soil pH was about 6.0 across treatments and over time declined to between 3.7 under manure to 4.5 under maize stover. The application of mineral N further acidified most treatments (Fig 2).

### 3.2 Temporal effects on fungal community structure and diversity

*Ascomycota* and *Basidiomycota* were the main phyla identified in all the treatments across the three-time point. At T1, *Ascomycota* recorded the higher frequencies > 93% across the organically amended treatments. However, no fungi were detected under the control at T1. At T2, *Basidiomycota* increased from < 6% to 18–22% while *Ascomycota* declined to 72–78% under *Crotalaria* and maize stover. Manure also housed *Chytridiomycota* at 10% frequency thus increasing community diversity (Hʹ = 0.78) (Table 2). However, at T3 *Basidiomycota* increased to 34% while *Ascomycota* declined to 66% under manure while both phyla remained unchanged in other treatments (Table 2).

**Table 2:** Dynamics of selected taxonomical classes in time at Domboshawa in 2015/16 rainfall season.

	T1				T2				T3			
	Crot	Man	MzStv	Cntl	Crot	Man	MzStv	Cntl	Crot	Man	MzStv	Cntl
Phyla count	2	2	2	nd	2	3	2	2	2	2	2	2
Hʹ	0.16	0.15	0.23	nd	0.53	0.78	0.06	0.3	0.47	0.64	0.31	0.25
Trend	Asc _(96%)_ > Bas _(4%)_	Asc _(96%)_ > Bas _(4%)_	Asc _(94%)_ > Bas _(6%)_	nd	Asc _(78%)_ > Bas _(22%)_	Asc _(72%)_ > Bas _(18%)_ > Chy _(10%)_	Asc _(99%)_ > Bas _(1%)_	Asc _(91%)_ > Bas _(9%)_	Asc _(82%)_ > Bas _(18%)_	Asc _(66%)_ > Bas _(34%)_	Asc _(91%)_ > Bas _(1%)_	Asc _(93%)_ > Bas _(7%)_
Class count	5	4	7	nd	5	7	3	6	4	4	6	4
Hʹ	0.93	0.96	0.73	nd	1.51	1.60	0.73	0.97	1.10	0.97	1.08	1.2
Trend	Dot _(69%)_ > Eur _(16%)_ > Sor _(12%)_	Dot _(54%)_ > Sor _(38%)_> Eur _(7%)_	Eur _(82%_) > Dot _(9%)_ > Sor _(3%)_	nd	Dot _(29%)_ = Sor _(30%)_ > Tre _(13%)_ > Ust _(8%)_	Dot _(31%)_ = Eur _(32%)_ > Tre _(15%)_ > Chyt _(11%)_	Dot _(61%)_ > Sor _(37%)_	Eur _(69%)_ > Dot _(20%)_ > Sor _(3%)_	Dot _(47%)_ > Sor _(34%)_ > Tre _(17%)_	Dot _(56%)_ > Tre _(33%)_ > Eur _(8%)_	Eur _(67%)_ > Dot _(14%)_ > Sor _(10%)_	Eur _(18%)_ > Dot _(38%)_ > Sor _(39%)_

Figures in parentheses are relative proportions; Crot = *Crotalaria*; Man = Manure; MzStv = Maize stover; Cntl = Control; Asc = *Ascomycota*; Bas = *Basidiomycota*; Chy = *Chytridiomycota*; Dot = *Dothideomycetes*; Eur = *Eurotiomycetes*; Sor = *Sordariomycetes*; Tre = *Tremellomycetes*; Ust = *Ustilaginomycotina Incertae sedis*; Chyt = *Chytridiomycete;* Hʹ = Shannon-Wiener diversity index, nd = not detected.

Focusing on classes, *Sordariomycetes*, *Eurotiomycetes*, and *Dothideomycetes* had the highest contribution to the *Ascomycota* while *Agaricomycetes* and *Tremellomycetes* were most abundant classes of *Basidiomycota*. At T1, 7 classes were identified across treatments with maize stover having all classes followed by *Crotalaria* with 5 and manure with 4. *Eurotiomycetes*, *Dothideomycetes*, *Sordariomycetes*, and *Tremellomycetes* were common amongst the three treatments but quality determined relative proportions (Table 2). Under *Crotalaria*, *Dothideomycetes* had a peak frequency of 69% followed by *Eurotiomycetes* and *Sordariomycetes*. Similarly, under manure, *Dothideomycetes* dominated (54%) followed by *Sordariomycetes* and *Eurotiomycetes*. However, *Eurotiomycetes* dominated under maize stover with 82%. At T2, under *Crotalaria*, *Dothideomycetes* significantly declined to 29%, while *Sordariomycetes*, *Tremellomycetes*, and *Ustilaginomycotina Incertae sedis* increased to 30%, 13% and 8%, respectively (Table 2). Under manure, *Dothideomycetes* and *Sordariomycetes* significantly declined to 30% and 9%, respectively. Simultaneously, *Eurotiomycetes* and *Tremellomycetes* significantly increased coupled with stimulation of three new classes. An overall reduction in fungal classes to three was observed under maize stover at T2 which was characterized by an increase in *Dothideomycetes* and *Sordariomycetes* to 61% and 37%, respectively. Simultaneously, *Eurotiomycetes* dropped from 82% to 1%. On the other hand, the control was dominated by *Eurotiomycetes* (69%) and *Dothideomycetes* (20%). *Dothideomycetes* attained the highest frequency under *Crotalaria* (47%) and manure (57%) both being an increase at T3 while *Eurotiomycetes* had the highest percentage (67%) under maize stover. A significant ~ 50% gain in *Tremellomycetes* frequencies was noted under manure at T3 (Table 2).

### 3.3 Fungal species abundance and diversity under the different treatments within the season

Amended soil provided a unique environment for housing a community of fungi. At T1 *Exophiala sp SST 2011*, *Cladosporium perangustum* and *Cladosporium sp 4 MU 2012* were common species across treatments (S1–S3 Figs). Manure had the highest frequency of *Humicola fuscoatra* (41%) and exhibited higher community diversity (Hʹ = 2.14). *Exophiala sp SST 2011* had the highest frequency of 89% under maize stover while *Cladosporium perangustum* and *Cladosporium sp 4 MU 2012* had higher frequencies of 27% and 21% under *Crotalaria*, respectively. *Crotalaria* presented the second most diverse community (Hʹ = 1.98) with eleven fungal species while maize stover only had six species making it the least diverse community (Hʹ = 0.52) (S3 Fig).

At T2, manure housed 23 species (Hʹ = 2.69) followed by *Crotalaria* with 19 species (Hʹ = 2.54) and maize with 15 species (Hʹ = 2.18). *Humicola fuscoatra*, *Aureobasidium pullulans*, *A*. *leucospermi*, and *A*. *pullulans var subglaciale* vanished under manure, while under maize, *Aureobasidium* and four new *Cladosporium* species emerged (S1–S3 Figs). *Humicola fuscoatra* had the highest frequency of 39% under maize stover. *Phoma glomerata* was common across treatments but highest frequency (12%) was under *Crotalaria*. *Exophiala sp SST 2011* had peak frequencies under manure (32%), *Crotalaria* (16%), and was absent in maize stover. The control had the least community diversity (Hʹ = 1.03) but notably had the highest frequency of *Exophiala sp SST 2011* (75%) than all amended treatments.

At T3, *Crotalaria* and control housed the highest number of species (17) followed by manure (12) and maize stover (9). *Crotalaria* had the highest community diversity (Hʹ = 2.50), the least was in maize stover (Hʹ = 1.05) while manure (Hʹ = 2.21) and control (Hʹ = 2.22) were similar. *Phoma glomerata* and *Cryptococcus terreus* were common species across treatments, but higher frequencies of 18% and 14%, respectively, were observed under *Crotalaria* treatment (S3 Fig). Manure and *Crotalaria* housed five common species including *Alternaria alternata*, *A*. *longipes*, *Cryptococcus sp AL V*, and *C*. *aerius* of which higher frequencies were noted under manure. *Cryptococcus terreus* showed the highest frequency of 22% followed by the two *Alternaria* species with 16% under manure. *Exophiala SST 2011* resurged and dominated (75%) under maize stover while it declined under the control from 75% to 8%. Apart from *Humicola fuscotra* (40%) and *Leptosphaerulina chartarum* (9%), the control also housed eight unique species which were absent in organically amended treatments.

**Fig 3:**
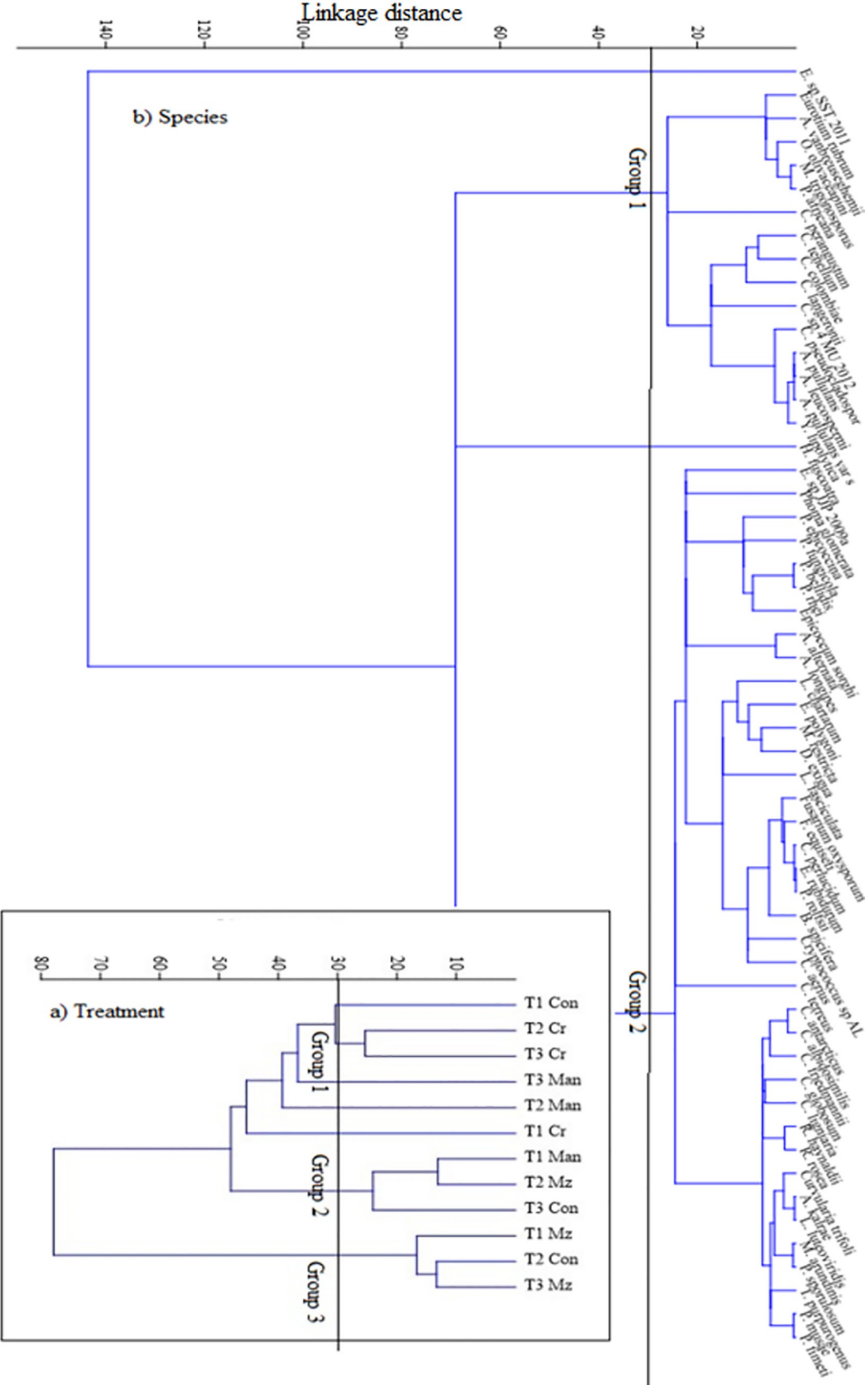
Dendrogram of the hierarchical cluster analysis indicating similarity in a) Treatments at each sampling points (Cr = *crotalaria*; Man = manure; Mz = maize stover; Con = control) and b) fungal species communities associated with soil samples from the three organically amended soils without N at the three-time points.

Cluster analysis identified three groups and four unique treatments at a distance of 30 (Fig 3A). Distinct treatments were control at T1, *Crotalaria* at T1, manure at T2 and T3. The first group indicated similarities amongst *Crotalaria* treatments at T2 and T3. In the second group, manure at T1 and maize stover at T2 were similar. Finally, the control at T2 and maize stover at T3 were similar in the third group (Fig 3A). Furthermore, species cluster analysis identified two groups and two unique species (*Exophiala sp SST 2011* and *Humicola fuscoatra*) at a distance of 30 (Fig 3B). *Cladosporium perangustum*, *Cryptococcus sp L V*, *Cladosporium sp 4 MU 2012*, *Cryptococcus terreus*, *Epicoccum sp JJP 2009a*, *Phoma glomerata*, *Epicoccum sorghii*, *Leptosphaerulina chartarum*, *Lecythophora fasciculata*, *Lecythophora specifera*, *Cladosprorium albudosimulis*, *Curvularia trifolii* and *Talaromyces purpurogenus* were outstanding species within the two main groups (Fig 3B).

### 3.4 The effects of pre-season N, ammonium nitrate and soil available P on fungal community composition

The addition of ammonium nitrate affected the dynamics and community structure differently at various taxonomical levels across the time points (S1–S3 Figs). No fungi were detected under the control + N at T1. At T1, pre-season N stimulated a wide range of *Cryptococcus* species, *Penicillium raper*, *Aspergillus reptans*, and *Eurotium rubrum* under *Crotalaria*. *Humicola fuscoatra* significantly increased from 10% to 68% while the frequencies of *Exophiala sp SST 2011* and *Cladosporium perangustum* were reduced. Furthermore, *Ophiostoma olivaceapini*, *Cladosporium tenellum*, *C*. *colombiae*, and *Epicoccum sp JJP 2009a* were absent under *Crotalaria* + N in December thus being the least diverse community (Hʹ = 1.39). Stimulation of *Arthroderma vanbreuseghemii*, *Ophiostoma olivaceapini*, and *Malassezia restricta* was noted under manure + N. Besides stimulation, the magnitude of *Exophiala sp SST 2011* and most *Cladosporium* species increased under manure + N. Contrary to *Crotalaria* + N, *Humicola fuscoatra* was significantly reduced from 41% to 8% under manure + N. However, eight species (including *Alternaria*) that were identified under manure were suppressed under manure + N thus becoming the second most diverse community (Hʹ = 2.10). Maize stover + N supported the most diverse community (Hʹ = 2.20) with nine emerging species and increased magnitude of *Cladosporium sp 4 MU 2012* from 2% to 12%. However, *Exophiala sp SST 2011* frequency was significantly reduced from 89% to 3% under maize stover + N at T1.

At T2, the addition of N under *Crotalaria* introduced a new regime of species (*Curvularia trifolii* (12%), *C*. *gladioli* (3%), *Mortierella parvispora* (6%), *M*. *polycephala* (4%), *M*. *humilis* (1%), and *Cordyceps chlamydosporia* (1%)). Application of N under *Crotalaria* also increased the frequencies of *Cryptococcus terreus* (7% to 27%), *Phoma glomerata* (2% to 17%), *Humicola fuscoatra* (7% to 13%) and *Phoma fungicola* (2% to 13%). However, most of the 19 fungal species under *Crotalaria* (Hʹ = 2.69) were suppressed to 11 species under *Crotalaria* + N (Hʹ = 2.04). Twelve new fungal species emerged following the addition of N under manure with *Cryptococcus terreus* being outstanding as it attained the highest frequency of 44%. However, 15 species inclusive of four *Cladosporium*, two *Alternaria*, and two *Cryptococcus* species were suppressed following the addition of N under manure (Hʹ = 1.96). At the same time, the magnitude of persistent species was reduced, notably *Exophiala sp SST 2011* was reduced to 21%. Addition of N under maize stover, reduced the frequency of *Humicola fuscoatra* (39% to 1%) while *Phoma glomerata*, *four Cladosporium* and three *Aureobasidium* species were suppressed. Fourteen new species emerged following the addition of N under maize stover, with *Curvularia trifolii*, *Coniochaeta ligniaria* and *Cryptococcus terreus* showing 35%, 17% and 11%, respectively. Similarly, application of N under the control stimulated 15 new species in the community inclusive of *Curvularia trifolii* (37%), *Coniochaeta ligniaria* (13%), and *Hamigera insecticola* (6%). However, application of N under the control inhibited five species including *Exophiala sp SST 2011* and *Cladosporium perangustum* while the magnitude of other species was reduced. Overall, the number of species doubled to 18, and community diversity (Hʹ) significantly increased to 2.28 following the addition of N to the control.

At T3, maize stover + N was the most diverse (Hʹ = 2.81) with 22 species followed by control + N (Hʹ = 2.77) with 23 species, then manure + N (Hʹ = 2.56) with 18 species and the least diverse was *Crotalaria* + N (Hʹ = 1.85) with twelve species (S1–S3 Figs). The addition of N under *Crotalaria* increased the magnitude of *Humicola fuscoatra* to 46% and *Leptosphaerulina chartarum* to 11% when compared to *Crotalaria*. *Talaromyces purpurogenus* and *Fusarium cf equiseti MY 2011* were two new species identified due to N application under *Crotalaria*. However, N significantly reduced *Phoma glomerata* to 9% and *Cryptococcus terreus* to 4% while several species (inclusive of *Alternaria alternata*, *A*. *longipes*, *Cryptococcus aerius* and *C*. *sp AL V)* vanished. Under manure + N *Cryptococcus terreus* maintained its higher frequency of 22% followed by *Humicola fuscoatra* with 17%. Furthermore, N increased *Phoma glomerata* to 3% while *Exophiala sp SST 2011* was reduced to 1%. *Phoma epicoccina*, *Alternaria alternata*, *A*. *longipes*, *Cryptococcus aerius* and *C*. *friedmannii* vanished while fourteen new species were stimulated under manure + N. Six species inclusive of *Exophiala sp SST 2011* vanished while *Lecythophora fasciculata* was reduced from 7% to 5% under maize stover + N. *Cryptococcus terreus* and *Microsphaeropsis arundinis* frequencies increased to 8% and 6%, respectively following N addition to maize stover treatment. Furthermore, the application of N under maize stover stimulated fifteen new species inclusive of five *Cladosporium*, two *Curvularia*, and two *Hamigera* (S3 Fig). Application of N under the control stimulated sixteen new species with *Epicoccum nigrum* exhibiting peak frequency of 20%. On the other hand, ten species inclusive of two *Fusarium* and two *Phoma* vanished following the addition of N under the control. Significant reduction of *Exophiala sp SST 2011* from 8% to 1% and *Humicola fuscoatra* from 40% to 6% was noted following the application of N under the control.

**Fig 4:**
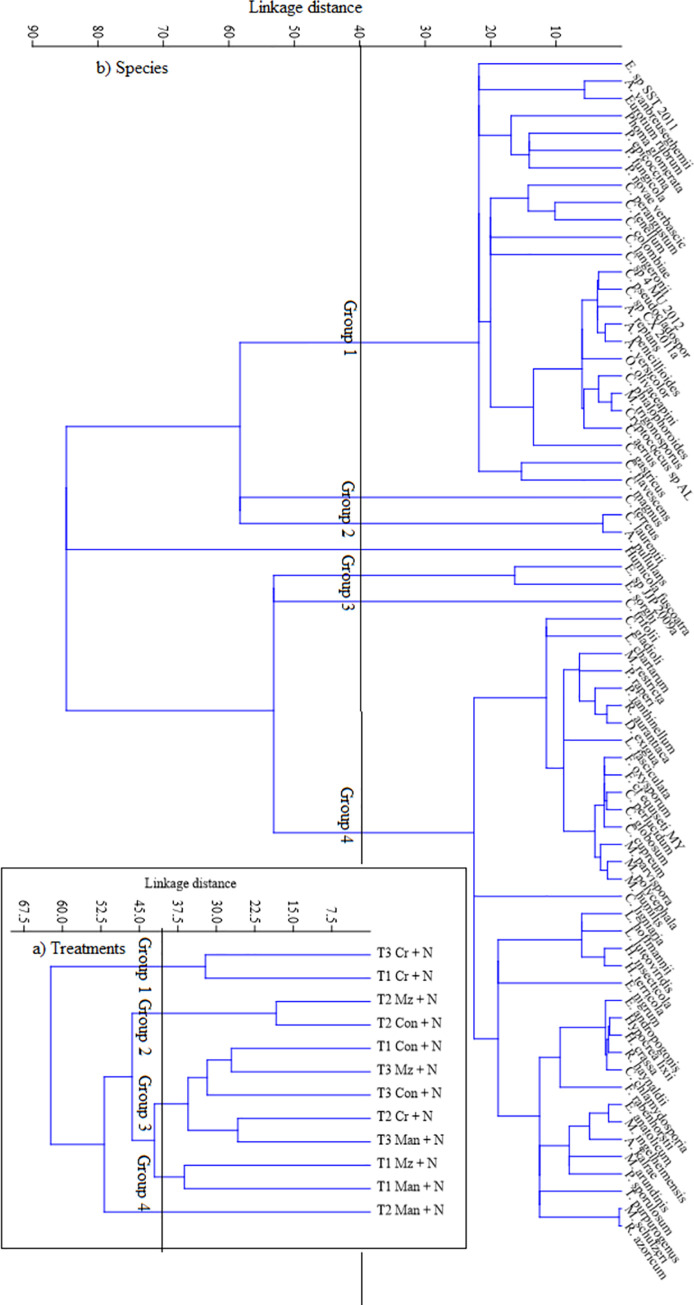
Dendrogram of the hierarchical cluster analysis indicating similarity in a) Treatments at each sampling points (Cr = *crotalaria*; Man = manure; Mz = maize stover; Con = control) and b) fungal species communities associated with soil samples from the three organically amended soils with N at the three-time points.

**Fig 5:**
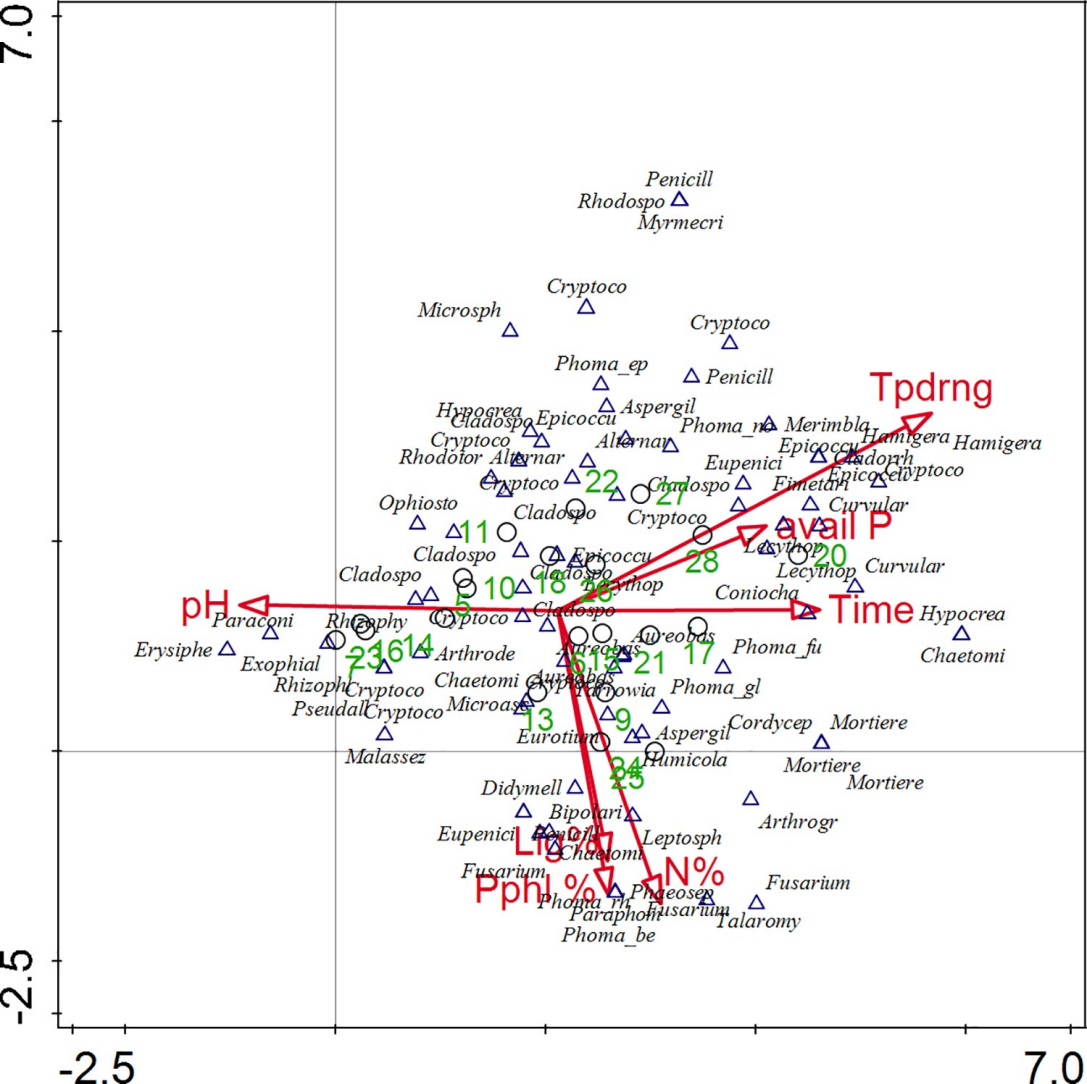
Detrended correspondence analysis (DCA) diagrams of species, environmental variable and samples. The direction of the vectors indicates the direction of maximum change of that variable, whereas the length of the vector represents how well the parameter explains the distribution of the data. Samples are numbered in green, external variable (avail P = soil available phosphorus, Lig% = Lignin content, N% = total N in organic amendment, pH = soil pH, Pphl% = polyphenol content, Time = season time, Tpddrng = mineral nitrogen application).

Cluster analysis identified four groups and one unique treatment (manure +N at T2) at a distance of 40 (Fig 4A). The first group indicated similarities among *Crotalaria* + N treatments at T1 and T3. The second group had maize stover + N and the control + N at T2. The majority of the treatments were in the third group. Maize stover + N and manure + N at T1 were assigned to the fourth group (Fig 4A). Four groups of fungi and three unique species (*Humicola fuscoatra*, *Cryptococcus terreus* and *Curvularia trifolii*) were identified at a distance of 40 following species cluster analysis (Fig 4B). *Phoma glomerata*, *Exophiala sp SST 2011*, *Cladosporium perangustum*, *Ophiostoma olivaceapini*, *Cryptococcus gastricus*, *Lecythophora fasciculata*, *Coniochaeta ligniaria*, *Fimetariella rabenhorstii* and *Epicoccum nigrum* were also outstanding species within the main groups (Fig 4B). Overall, most species were positively correlated to mineral N application, soil pH, and soil available P. Seasonal time also had species responding to it. Organic resources quality attributes explained fungal species identified under *Crotalaria* and the control (Fig 5). Application of mineral N was the most influential variable followed by soil pH, resource total N > seasonal time = resource polyphenols and the least factors being resource lignin and soil available P (Fig 5). However, an interactive-forward test indicated that the application of mineral N and soil available P significantly (P < 0.05) affected fungal species (S1 Table).

## 4 Discussion

### 4.1 The dynamics of fungal population and community diversity

December sampling was done during a wet and warm period that promoted fungi proliferation, a trend similar to increased microbial activity noted by Amato and Ladd [37]. That sampling was also within the 5–15 days stipulated by Chen and Ferris [38] for full fungi colonization which increased precision of fungal diversity capture. Phosphorus, potassium and sulphur were adequately supplied to sustain the system and thus the fungal dynamics would be linked to resource quality, organic matter and subsequent N released. The disparity between the applied and soil available P suggest that most of the P was being immobilized by microbes. All the fungi noted in this study were able to survive in a wide range of soil pH (3.6–6.5) and would not impact major shift in community structure which concur with other studies [39, 40]. The increase in soil available P and community diversity suggest that phosphorus was critical in stimulating fungal growth.

The presence of *Ascomycota* and *Basidiomycota* under *Crotalaria*, manure, and maize stover treatments support other studies that indicated both as the two common groups of fungi playing a primary role in decomposition of organic matter in soil [22, 41, 42]. Subsequent changes in phyla are probably linked to stabilization process, influence of soil conditions and availability of substrate for utilization [40, 43, 44]. Once the organic amendments were applied coupled with wet conditions, fungal succession started for example under manure as *Basidiomycota* decreased from 7% to 3% while *Zygomycota* vanished. Similarly, under manure, *Basidiomycota* continued to rise while *Ascomycota* decreased as decomposition progressed. This could be attributed to the accumulation of lignin and cellulose that is utilizable by *Basidiomycetes* [45]. Fungal succession observed under the different quality organic resources in this study was comparable to dynamics under woody debris that involved *Zygomycetes* first, followed by *Ascomycetes* and lastly *Basidiomycetes* [45]. However, the continued increase in *Ascomycota* and decrease in *Basidiomycota* indicate the presence of drought-tolerant melanized *Ascomycota* decomposers that predominate over *Basidiomycota* [46]. This adaptation ensures survival under the harsh conditions (e.g., dry spells and dry season from April to October) and becomes even useful in the wet season. The significant drop in *Dothideomycetes* under both *Crotalaria* and manure accompanied with an increase in *Sordariomycetes*, *Tremellomycetes*, and *Ustilaginomycotina Incertae sedis* under *Crotalaria* confirms fungal succession as the wet season progresses. The combined persistence of *Humicola fuscoatra* and *Phoma glomerata* is likely to be the driver of net mineralization under *Crotalaria*. The high frequency of *Cladosporium perangustum* and *Cladosporium sp 4 MU 2012* under this high quality organic soil amendment suggests that both species are first colonizers tapping on simple sugars. Besides *Cladosporium* species, *Humicola fuscoatra* was also an outstanding first colonizer under *Crotalaria* and manure and this can be linked to the high soil available N [25, 47]. Similarly, Taylor and Sinsabaugh [45] highlighted the influence of carbohydrate, phenolic composition, C: N: P ratios as the basis for differentiation and successional patterns in fungi community.

Application of Sprengel-Liebig law of the minimum [48], will indicate that N was probably the most limiting nutrient affecting the fungal diversity compounded by source of the nutrients and mode application. *Crotalaria* is known to decompose rapidly under wet and warm conditions [25, 49], subsequently promoting high frequency of *Dothideomycetes* 69% followed by *Eurotiomycetes* (16%) and *Sordariomycetes* 12% due to released trigger molecules. According to DeNobili et al. [50], trigger molecules are diverse, soluble, low molecular weight and diffusible molecule released by organic amendments that activates a dormant cell in preparation for available resource. High-quality organic resources release several trigger molecules [50], due to a high percentage easily degradable compounds such as simple sugars, proteins and low percentage of complex substrates (cellulose and lignin) thereby promoting proliferation of opportunistic sugar fungi [43]. This therefore suggests that manure and maize stover have less trigger molecules and higher proportions of undegradable compounds to promote colonization by sugar-loving fungi.

The attainment of almost equivalent *Dothideomycetes* frequencies under manure (54%) and *Crotalaria* (69%) in December is linked to readily available nutrients under manure and similarity of released trigger molecules under both treatments. According to Snajdr et al. [51], both *Dothideomycetes* and *Sordariomycetes* are first colonizers mostly found as endophytes on dead leaves which is similar to our finding. At the same time, manure promoted more growth of *Sordariomycetes* than *Eurotiomycetes* which might be linked to presence of unique substrates (inorganic and organic). To cement that, quality was a major contributing factor is shown by dominance of *Eurotiomycetes* under maize stover which was of lower frequency under both *Crotalaria* and manure in December.

The persistence of *Humicola fuscoatra*, *Phoma glomerata* coupled with an increase in *Phoma fungicola*, *Lecythophora fasciculate*, *and Cryptococcus terreus* in February (mid-season period) confirm that high-quality *Crotalaria* can sustain all fungal species plus the growing crop. Continuous supply of soluble carbon and trigger molecules from decomposition proliferated and maintained sugar fungi within the season [6, 43]. According to Botha [52], the genus *Cryptococcus* utilizes simpler by-products of decomposition from complex plant structural components thereby promoting the release of soluble carbon which drives persistence and increase magnitude of some fungi. We argue that the species diversity might not be important as species function in a system since results from this study indicated that while *Crotalaria* and the control treatment had the same number of species, it is without doubt that treatment influence on productivity will never be the same. Likewise, we also noticed similar diversity under manure and control yet research has shown comparatively higher crop yields under manure suggesting that these type fungi may not be directly influencing crop productivity. We therefore propose application of high-quality resources to ensure high diversity of purposeful species to aid organic matter decomposition from enhanced plant growth.

### 4.2 The effect of ammonium nitrate and available P on fungi community structure

Manipulation of soil fertility with external fertilizers affected fungal dynamics and community structure within the season. Given that only phosphorus, sulphur, and potassium were co-applied with organic resources, points to pre-season soil N and decomposition as source of N. Sampling was done 7 days after the application of organics such that most available N is from pre-season decomposition of roots or soil organic matter [53] except for high-quality which decompose rapidly. The highlighted P immobilization in December suggests that it was also a limiting nutrient for fungal proliferation. The warm and wet conditions promoted decomposition of organic amendments depending on their quality [49] consequently creating different habitats for fungal colonization. Besides trigger molecules, the sudden wetting of dry soil under the various treatments released carbon and nitrogen from a pulse in soil C and N mineralization “birch effect” [50, 54, 55]. That ‘birch effect’ plays a major role in structuring fungal community at season onset as both nutrients (C and N) are critical for microbial survival. The interaction of SOM build-up [5] and birch effect under low quality resource also shapes fungal diversity. The pre-season N and available carbon within the treatments contributed to community composition differences and linked to organic resource quality.

Quality of organic resource applied and the maize root system can explain the fungal composition differences noted under the three organic resources with N. Under maize stover this pre-season N was most critical in community composition as it was able to stimulate nine new species, increase *Cladosporium sup 4 MU 2012* frequency. This can be termed “indirect manipulation” of the fungal community structure. The high frequency of *Humicola fuscoatra* (69%) under *Crotalaria* + N could be in response to the availability of a high available N and simple sugars released from residual abundant dead roots (previous maize crop and dry season weeds), the little SOM and the current resource applied [5, 25]. The reduction in *Exophiala sp SST 2011* and *Cladosporium perangustum* frequencies indicates the detrimental effect of available N and influx of simple sugars (deprived of cellulose and lignin), under *Crotalaria* + N. The direct detrimental effect of ammonium nitrate was confirmed by the vanishing of different species and reduction of community diversity across treatments and over time [42, 56, 57].

The results also indicate that addition of N under the high-quality resource led to stimulation of new species and increased magnitude of *Cryptococcus terreus*, *Curvularia trifolii*, *Humicola fuscoatra*, *Phoma glomerata*, and *P*. *fungicola*, which could be nitrophylic fungi. According to Morrison et al. [58], nitrophylic fungal species are more adapted to high soil N particularly following application of high-quality resources or N fertilizers [29, 59]. Fungi proliferation was high when both N and P were adequate otherwise, if one (N or P) was in short supply that multiplication might be limited. Increase in soil available P from February onwards across treatments was critical for fungi as indicated by interactive-forward test that identified soil available P as a key factor. Similarly, cereals respond to N fertilizer only when P deficiency has been corrected [49]. The N priming effect and P availability under manure led to twelve new species being stimulated coupled with increased magnitude of *Cryptococcus terreus* and significant reduction of *Exophiala sp SST 2011*.

Increased decomposition of applied organics, roots and dead microbes promote influx of simple sugars, N and available P causing adaptable fungi species to manifest or proliferate while others declined. Different fungi have substrate preferences [45] and will proliferate under environment with preferred substrates, and limited by non-preferred substrates. The concentration of the substrates (preferred or non-preferred) could explain *Exophiala sp SST 2011* dynamics. Since decomposition is at its slowest rate in the control, a nutrient starved system [60], any little N and P added would significantly jump-start the fungal activity, particularly nitrophylic species to increase the diversity. According to DeNobili et al. [50], most microbes under such starved systems would be in a “metabolic alertness” mode such that any trigger molecules would induce significant impact in microbial growth. The benefits of high-quality resources is in supplying trigger molecules and substrates (substantial food event) for fungal growth [50] and proliferation is evident under *Crotalaria* + N. Nitrogen primed the release of more trigger molecules and substrates that subsequently increased magnitude of *Humicola fuscoatra* and *Leptosphaerulina chartarum* while stimulating *Talaromyces purpurogenus* and *Fusarium cf equiseti MY 2011*. Such dynamics shown under a high-quality resource is evidence of fungal proliferation despite major drawback of N losses previously documented [29, 61]. Application of ammonium nitrate under medium to low quality resource and the control was key in offsetting immobilization [38] thereby promoted availability of substrates for fungal stimulation, increasing magnitude of nitrophylic fungi while suppressing *Exophiala SST* 2011.

### 4.3 Implications of fungal dynamics in cropping systems

We noted that P was immobilized following co-application of fertilizers and organic resources during period when the nutrient is at peak demand by the growing crop and this will evidently negatively impact crop development. To avoid shortage of P at crop establishment, hidden hunger and manifestations of P deficiency symptoms, judicious amounts should be applied in sandy soils to counteract any impending fungal P immobilization. Without proper root development, nutrients and water uptake will limit crop development and subsequently reduce the expected yield. From January, crop nutrient uptake would be a competitor for fungi [62]. However, when nutrients are adequate for both crops and soil fungi for example under *Crotalaria* competition will be minimum or none. February coincides with period of high nutrient demand for crops (a stronger competitor for fungi) and having low fungal diversity under the control is the cause of poor crop stand and low yields. For crop production, there is need for high fungal diversity to aid decomposition and supply of nutrients. However, quality of organic resource is a critical factor in managing the diversity thus the need to utilize high-quality resources for crop production. Low-quality nutrient resources promote the presence of *Eurotiomycetes* such as *Exophiala sp SST 2011* which is linked to N immobilization thereby limiting crop development [38, 63]. Those periods of immobilization or reduced supply of nutrients within the cropping season are the main reasons for having low crop yields under cereal residues, conservation agriculture, and poor-quality manure in most parts of SSA [64, 65]. Addition of N to low-quality resources or mixing them with medium to high quality resources is therefore recommended to reduce the magnitude of *Exophiala sp SST 2011* and promote species that drive net mineralization. Overall, the ratio of *Dothideomycetes*: *Eurotiomycetes* in soils following application of organic amendments is likely to be a good indicator of net mineralization or immobilization.

## 5 Conclusion

Organic resource quality influenced the fungal diversity species as a function of time. Our results showed that availability of the critical nutrients, N and P as dictated by the mineralization and immobilization regimes of the nutrients from the different organics was key for the survival, proliferation and succession of the different fungi. Soil fungi were adaptable to a wide range of soil pH. Birch effect, a results of pre-season N availability influenced the fungal community structure at T1 while ready sources of available N, in this case ammonium-nitrate fertilizer were critical at T2 and T3, being more pronounced under medium to low-quality organic resources. To compensate for fungal immobilization that threatens maize development and yield, it might be critical to increase P application rate beyond 16 kg ha^-1^. Low-quality nutrient resources promoted the presence of *Exophiala sp SST 2011* which is linked to micro-environments typified by N immobilization. Co-application of medium to high quality organic with inorganic resources shows promise in sustaining and supporting belowground fungal populations, a key driver in soil nutrient supply processes.

## Supporting information

S1 Fig Fungal species dynamics under *Crotalaria* with and without N at Domboshawa during 2015/16 season. (DOC)

S2 Fig Fungal species dynamics under manure with and without N at Domboshawa during 2015/16 season. (DOC)

S3 Fig Fungal species dynamics under maize stover with and without N at Domboshawa during 2015/16 season. (DOC)

S1 Table Detailed results of forwarding selection test analysis. (DOC)

S2 Table Minimum data set for soil available P. (XLSX)

S3 Table Minimum data set for soil pH. (XLSX)
