# Supplementary material for: Soil fungal community structure and seasonal diversity following application of organic amendments of different quality under maize cropping in Zimbabwe
Source: PLoS One. 2021 Oct 14;16(10):e0258227. doi: 10.1371/journal.pone.0258227 (PMC8516296; doi:10.1371/journal.pone.0258227)
Supplement: S1 Fig — (DOC) [file pone.0258227.s001.doc]

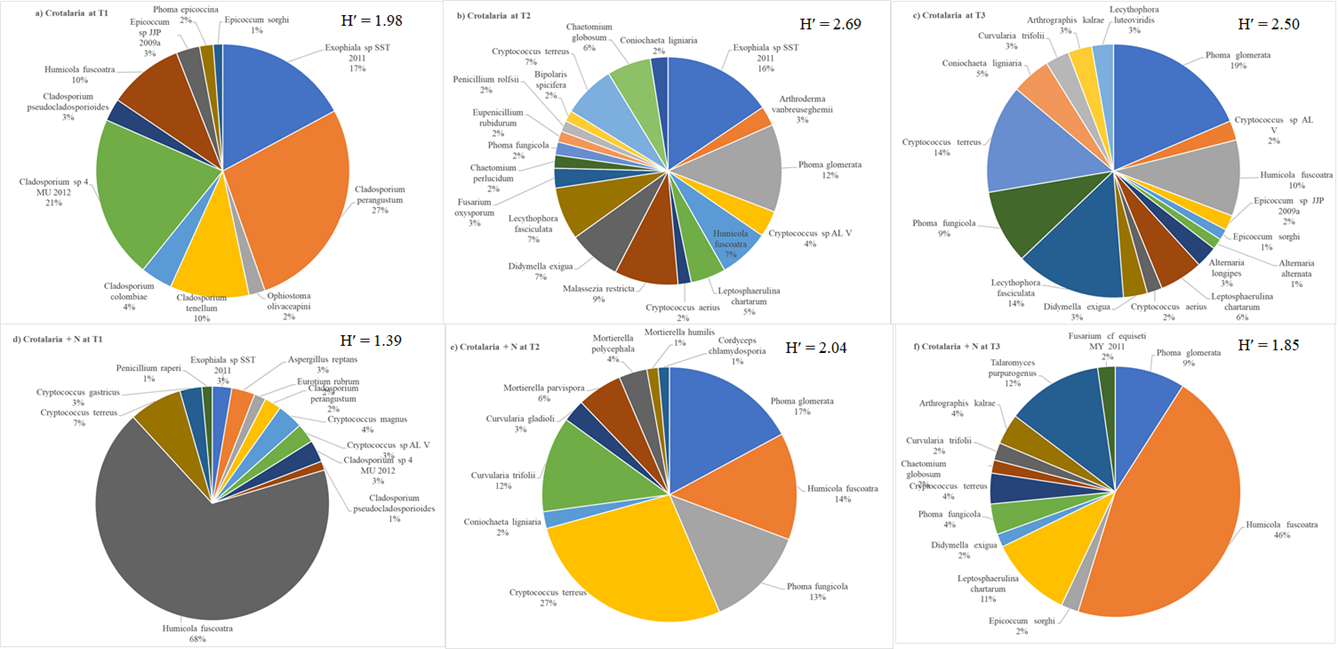


S1 Figure. Fungal species dynamics under *Crotalaria* with and without N at Domboshawa during 2015/16 season
