# Supplementary figures and images for: Soil fungal community structure and seasonal diversity following application of organic amendments of different quality under maize cropping in Zimbabwe
Source: PLoS One. 2021 Oct 14;16(10):e0258227. doi: 10.1371/journal.pone.0258227 (PMC8516296; doi:10.1371/journal.pone.0258227)

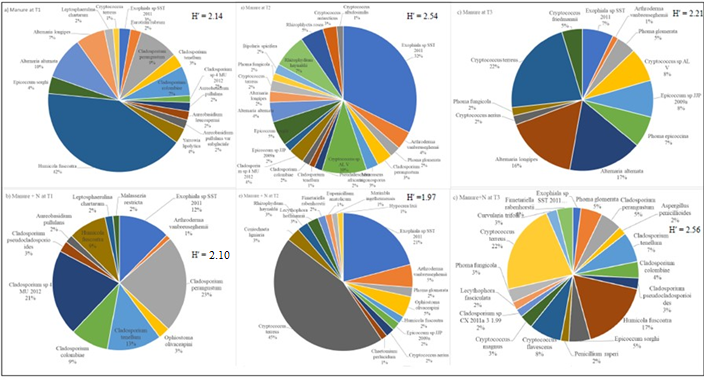


S2 Figure. Fungal species dynamics under manure with and without N at Domboshawa during 2015/16 season

Supplement: S2 Fig — (DOC) [file pone.0258227.s002.doc]
