# Supplementary material for: Soil fungal community structure and seasonal diversity following application of organic amendments of different quality under maize cropping in Zimbabwe
Source: PLoS One. 2021 Oct 14;16(10):e0258227. doi: 10.1371/journal.pone.0258227 (PMC8516296; doi:10.1371/journal.pone.0258227)
Supplement: S1 Table — (DOC) [file pone.0258227.s004.doc]

**Table S1. Detailed results of forward selection test analysis**

| **External variables** | **Explains %** | **Contribution %** | **pseudo-F** | **P** |
| --- | --- | --- | --- | --- |
| **Tpdrng** | 8.0 | 20.4 | 1.7 | 0.02 |
| **avail P** | 7.5 | 19.1 | 1.6 | 0.02 |
| **N%** | 5.9 | 14.9 | 1.3 | 0.156 |
| **Time** | 5.2 | 13.3 | 1.1 | 0.286 |
| **Lig%** | 4.9 | 12.4 | 1.1 | 0.383 |
| **Pphl %** | 4.5 | 11.4 | 1.0 | 0.52 |
| **pH** | 3.3 | 8.5 | 0.7 | 0.816 |

Tpdrng = mineral nitrogen application, avail P = soil available phosphorus, N % = total N in organic

amendment, Time = season time, Lig % = Lignin content, Pphl % = polyphenol content, pH = soil pH
